# Systemic Remedies in Diseases of the Teeth

**Published:** 1899-08

**Authors:** Leo Greenbaum


ARTICLE IV.

### SYSTEMIC REMEDIES IN DISEASES OF THE TEETH.

LEO GREENBAUM, M. D., D. D. S.

In compliance with the very courteous invitation of your committee I wish to present a short survey of systemic remedies which may be used in the treatment of diseases associated with the teeth. In doing this it is not my intention to enter into a discussion of the legal status or ethical propriety of dentists prescribing drugs for internal medication, but simply to point out the applicability of such medicines as adjuncts to local treatment. I will further limit my subject by considering only the systemic treatment of severe pains which frequently accompany dental diseases and which do not really yield to local treatment.

Among such conditions we find hypersensitive dentine. By hypersensitive dentine I mean a degree of sensitiveness in which the touch of an instrument produces a painful impression, and which interferes with the proper preparation of a cavity for the introduction of a filling; the use of a bur or excavator causing intense suffering to the patient. This condition is responsible, as we all know, for the wholesale neglect of the teeth on the part of the public. One experience is severe enough to strongly and permanently impress with dread of dental operations not only hysterical women but even strong men, and to cause them ever afterwards to neglect proper professional care of their teeth. Pathologists claim this hypersensitivity to be due to increased functional activity of the so called fibrillæ in dentine denuded of its protecting covering and thus exposed to external stimulation and irritation.

But this increase in the local irritability is not sufficient to account for the horror manifested by some of our patients at the sight of dental instruments, and which causes them instinctively to grasp the hand of the operator or to jerk the head back so as to avoid instrumental contact. This effect must fairly be attributed to causes other than the local condition. Therefore local treatment, such as the use of sharp burs, dehydration of the affected part by means of warm air, the application of alkalies or local anodynes, or agents which destroy the dentinal protoplasm—excellent in ordinary cases—is not sufficient to control these extreme ones.

The introduction of cataphoresis has helped very much to overcome such difficulties; but experience teaches us that patients who have once undergone the sufferings attendant upon the excavation of a cavity under such circumstances of hypersensivity are ever after rather difficult to manage before they can be induced to submit to the application of the electric current; and that in such cases it is not only better, but necessary, to produce some systemic effect by means of agents which either stimulate or diminish the perception of impression in the nerve-centres.

Various remedies have been suggested for this purpose, such as the inhalation of general anesthetics, chloroform and ether, in quantity sufficient to benumb sensibility; but there is decided objection to this mode of treatment on account of the disagreeable after effects liable to result and the danger to life lurking in these drugs.

The various coal-tar derivatives and the bromides have also been used; but the best results have been obtained by the use of the old-fashioned drug asafœtida. Asafœtida has a decided stimulating effect upon the nervous system, and produces a kind of pleasant intoxication which removes fear and overcomes nervousness. I have had several cases in which a patient on first consulting me was so overwhelmed with fear that it was impossible to attempt an examination; when the use of an explorer was out of the question, and even the mouth-mirror was carefully examined before its introduction into the mouth was permitted. In such cases good results have followed the administration of two three-grain asafœtida pills, the first one hour and the other a half hour before the time appointed for the operation. There is neither danger nor disagreeable after effects from the use of this drug, and it supports the patient's system and causes more ready submission to the operation.

Diseases of the pulp, such as the formation of pulp nodules, pulpitis, etc., are often associated with reflex pains in the face, side of the head, in the ear, etc., of intense character, entailing a great deal of suffering, and which require for their cure pulp devitalization. When this can be accomplished promptly the cause of the pain is removed and the patient relieved. But many cases present obstacles to the action of the devitalizing agent, as an inflammatory condition of the pulp arsenic will not act promptly, while in cases of pulp-nodules entrance to the pulp-chamber must first be secured. Occasionally it may be desirable to attempt pulp preservation. The consequent delay of the final cure would not satisfy the sufferer unless the neuralgic attack, for the treatment of which he consulted the dentist, were relieved. In such cases good results may be obtained from the internal administration of the coal-tar derivatives, such as acetanilide, phenacetin, and others. Experience in the use of these agents has demonstrated the fact that their effects are modified by individual peculiarities more frequently than any other group of medicines. I found that some patients were benefited by acetanilide, whereas upon others it had no effect, but phenacetin acted favorably; and in a similar manner all other drugs belonging to this group. This led me to the use of a combination of several of these medicines in one prescription, from which I obtained more universal good results than when using them singly. It seems that when in combination the therapeutic properties are acting conjointly in perfect harmony, while the toxic properties are more or less neutralized by each other; and even smaller doses of these analgesics when in combination are more powerful and more certain to alleviate pain than any one of them individually, and, above all, being free from the unpleasant symptoms frequently following a similar dose of any one of them. I have used the following prescription in many cases:

℞ Acetanilide, gr. viii;
Phenacetin, gr. xv;
Caffeine citrate, gr. xv.
Misce et ft. pulv. no. viii.
Sig.—One to be taken every two hours.

Since writing the above I have read an article in one of the medical journals in which a physician recommends the use of these combinations for reasons similar to those I have named. His prescription seems excellent, and I intend to try it at the first opportunity which may present itself.

℞ Antipyrin,
Phenacetin,
Quinine sulphate,
Powdered ginger, aa ʒ ss;
Caffeine citrate, gr. xv.
Mix and divide into fifteen capsules.
Sig.—One every two hours.

Doubtless every practitioner has had some experience with high-grade pericementitis, in which the tooth continues to throb and hammer with inflammation increasing in spite of the aconite and iodine applied to the gum, and in spite of the persistent application of drug after drug to the effected part until the mouth is blistered, the throat sore, taste temporarily destroyed, the patient made generally and wofully miserable, and the dentist, in sheer desperation, has sent his patient home with the assurance that he has used all known remedies and done all in his power; or, worse, has been guilty of malpractice in extracting the offending tooth.

In such cases good result may be obtained if in addition to the local treatment the following internal sedative is prescribed:

Potassii bromidi, ʒ ii;
Aquæ, f℥ ii. M.
Sig.—A teaspoonful every two hours.

Frequently these peridental troubles, despite all treatment, will run to an abscess; or before the case reaches us it may have gone beyond the point when resolution could be established. The pain associated with the formation of pus and the final formation of a fistula is very intense, in many cases making the patient quite feverish and generally depressed. To allow the patient to bear the intense suffering and steady torture accompanying degeneration, causing him to pace the floor in agony until the case is relieved by the usual discharge, is akin to barbarism. For many years in such cases I have given the patient a capsicum bag with instruction how to apply it, so as to hasten the opening of a fistula, suggesting that it be applied before retiring and that the following be taken :

Chloral hydratis, gr. xxv;
Potassii bromidi, ʒ i.
Misce et ft. pulv. no. iii.
Sig.—One to be taken before retiring.

Though I have used this almost universally in such cases during the past ten years, I cannot recall a single instance when the patient failed to spend a comparatively comfortable night, and in the morning pus was discharging through a fistulous opening. Choral is looked upon with disfavor by the medical profession on account of the depressing effect upon the heart which is attributed to it. But the manner in which I recommend my patients to take it—that is, before retiring—seems to obviate that danger. I can say with perfect assurance, in consequence of the experience I have had with the above prescription, that there is no danger in the dose given when taken in the manner prescribed; and that I have obtained more satisfaction and my patients more comfort in cases where its employment is indicated than from any other drug.

A word about capsicum bags. I do not want the proper kind to be confounded with the so-called capsicum plasters which are now so universally sold by druggists for the cure of toothache. The first are made by filling with powdered capsicum a bag one side of which is made of rubber to protect the cheek, the other of muslin to permit the fluids of the mouth to enter, dissolve, and act on the tissues covering the root or roots of the teeth against which these bags are placed. The second kind are not very strong and are really worthless for the purpose of counter-irritation.—*International Dental Journal.*

